# Vancomycin-Resistant *Enterococcus faecium* Bacteremia in a Tertiary Care Hospital: Epidemiology, Antimicrobial Susceptibility, and Outcome

**DOI:** 10.1155/2014/958469

**Published:** 2014-03-05

**Authors:** Regis G. Rosa, Alexandre V. Schwarzbold, Rodrigo P. dos Santos, Eduardo E. Turra, Denise P. Machado, Luciano Z. Goldani

**Affiliations:** Section of Infectious Diseases, Hospital de Clínicas de Porto Alegre, Universidade Federal do Rio Grande do Sul, Ramiro Barcelos 2350, 90630000 Porto Alegre, RS, Brazil

## Abstract

Vancomycin-resistant *Enterococcus faecium* (VREF) has emerged as a relevant multidrug-resistant pathogen and potentially lethal etiology of health care associated infections worldwide. The objective of this retrospective cohort study was to assess factors associated with mortality in patients with VREF bacteremia in a major tertiary referral hospital in Southern Brazil. All documented cases of bacteremia identified between May 2010 and July 2012 were evaluated. Cox regression was performed to determine whether the characteristics related to the host or antimicrobial treatment were associated with the all-cause 30-day mortality. In total, 35 patients with documented VREF bacteremia were identified during the study period. The median APACHE-II score of the study population was 26 (interquartile range: 10). The overall 30-day mortality was 65.7%. All VREF isolates were sensitive to linezolid, daptomycin, and quinupristin-dalfopristin. Linezolid was the only antimicrobial agent with *in vitro* activity against VREF that was administered to the cohort. After multivariate analysis, linezolid treatment (HR, 0.08; 95% CI, 0.02–0.27) and presence of acute kidney injury at the onset of bacteremia (HR, 4.01; 95% CI, 1.62–9.94) were independently associated with mortality. Presentation with acute kidney injury and lack of treatment with an effective antibiotic poses risk for mortality in patients with VREF bacteremia.

## 1. Introduction

Vancomycin-resistant* Enterococcus faecium* (VREF) is currently one of the most important etiologies of nosocomial infections worldwide, mainly due to its typical profile of multidrug resistance and tendency to cause severe infections in critically ill patients [1, 2]. Risk factors for developing a nosocomial VREF infection include prolonged hospitalization; hospitalization in long-term facilities, surgical units, or intensive care units; multiple courses of antibiotics; solid organ and hematopoietic stem cell transplantation; and presence of comorbidities such as diabetes, renal failure, or hemodialysis [3–6]. In the continuum of VREF infections, bacteremia is of special interest, given that overall mortality rates may reach values higher than 60% with an attributable mortality of around 40% [7–12]. Unfortunately, few data are available concerning factors associated with mortality in the context of VREF bacteremia in different institutions. Therefore, we conducted a study with the aim of assessing factors associated with mortality in patients with VREF bacteremia in the current practice of a tertiary referral hospital.

## 2. Methods

### 2.1. Study Design, Patients, and Settings

A retrospective cohort study was performed with all cases of documented VREF bacteremia identified between May 2010 and July 2012. The present study was conducted at Hospital de Clínicas de Porto Alegre (HCPA), a major tertiary referral hospital in Southern Brazil. The patients were identified by retrieval from the computerized database established by the Infection Control Center of HCPA. Bacteremia by VREF was defined as 2 positive results of 2 independent blood cultures from a patient with fever (body temperature ≥38°C). Blood isolates were identified according to standard techniques and Vitek2 (bioMérieux) [13]. VREF was defined as an *Enterococcus faecium* isolate with an MIC of vancomycin ≥32 *μ*g/mL by the Etest (bioMérieux) according to the standards of the CLSI. The analyses of clinical features, antibiotic susceptibility tests, and outcomes were focused on those patients with VREF bacteremia. Medical records of the patients who had VREF bacteremia between May 2010 and July 2012 were reviewed. Patients who had ever developed VREF bacteremia before the study period were excluded. If patients developed several episodes of VREF bacteremia during the study period, only the first episode was investigated.

### 2.2. Variables

Variables retrieved from a standardized case report form included demographics, underlying comorbidities, APACHE II score (Acute Physiology and Chronic Health Evaluation) at the first 24 hours following clinical signs of bacteremia, initial plasma C-reactive protein, initial serum albumin, presence of acute kidney injury (defined as decreases in glomerular filtration rate >50% or an increase in serum creatinine to ≥1.5 times baseline), and whether the infection was acquired in ICU or clinical ward. Data regarding antimicrobial therapy administered (e.g., type of antibiotic, time to antibiotic, and duration of treatment) were also analyzed. The main outcome of this study was all-cause mortality within 30 days from VREF bacteremia.

### 2.3. Antibiotic Susceptibility Test

MICs for daptomycin, linezolid, and quinupristin-dalfopristin were determined by the Etest (bioMérieux), according to the manufacturer's guidelines (AB Biodisk). Daptomycin, quinupristin-dalfopristin, and linezolid resistance was defined as an isolate with an MIC greater than 4 *μ*g/mL, 4 *μ*g/mL, and 8 *μ*g/mL, respectively [14, 15]. A suspension of each isolate in Mueller-Hinton broth, adjusted to the density of a 0.5 McFarland standard, was swabbed in three directions to ensure uniform growth onto Mueller-Hinton agar plates. The MIC was read where inhibition of growth intersected the *E*-test strip. When small colonies grew within the zone of inhibition or a haze of growth occurred around MIC endpoints, the highest MIC intersection was recorded.

### 2.4. Statistical Analysis

A Cox proportional hazards regression was performed to determine risk factors for 30-day mortality in patients with VREF bacteremia. All variables that had a *P* value <0.10 in a univariate analysis were included. In the multivariate model, independent variables were eliminated from the highest to the lowest *P* value but remained in the model if the *P* value was less than 0.05. Hazard ratios were estimated along with 95% confidence intervals. Kaplan-Meier curves were used to calculate the time-dependent occurrence of death; the log-rank test was used for comparisons between groups. The software used for the statistical analysis was STATA version 12 (StataCorp LP, USA).

### 2.5. Ethics

The study was approved by the institutional review board of Hospital de Clínicas de Porto Alegre.

## 3. Results

In total, 35 patients with VREF bacteremia were evaluated during the study period. As shown in Table 1, the overall mean age of the study cohort was 46 years and 60% were male. Subjects with malignant neoplasm comprised 45.7% of the study population; hematologic malignancies accounted for most cases of cancer. Other important underlying comorbidities found were cirrhosis (11.4%) and diabetes mellitus (8.5%). All cases of VREF bacteremia were acquired after 48 hours of hospitalization (62.8% acquired in the intensive care unit and 37.2% acquired in the clinical ward). The median APACHE II value of all study patients was 26.0.

All VREF isolates had a vancomycin MIC ≥256 *μ*g/mL. The most common antibiotics initially administered to patients were vancomycin (48.5%), meropenem (42.8%), and piperacillin-tazobactam (14.2%). Linezolid was the only antimicrobial agent with *in vitro *activity against VREF that was administered to the cohort; 26 subjects (74.2% of the study population) were treated with linezolid (88.4% were treated via intravenous route; the remainder were treated via enteral route). The median time to linezolid treatment was 3 days (interquartile range [IQR]: 2 days). The median duration of linezolid treatment was 9.5 days (IQR: 7 days). The antibiotic schemes administered to the 9 patients that did not receive linezolid were vancomycin monotherapy (2 cases), cefepime monotherapy (2 cases), imipenem-cilastatin + clindamycin (1 case), meropenem + vancomycin (1 case), meropenem + vancomycin + gentamicin (1 case), piperacillin-tazobactam + vancomycin (1 case), and meropenem + metronidazole (1 case). The main reason for withholding linezolid was the sudden clinical deterioration of patients, in the context of lack of empiric effective antimicrobial treatment against VREF, resulting in death before blood culture results (88.8% of cases). Acute kidney injury occurred at the onset of VREF bacteremia in 12 patients, of which 50% were treated with linezolid. As expected, the median APACHE II score was higher for patients with acute kidney injury in comparison with patients without acute kidney injury (28.1 [IQR: 8] versus 25.5 [IQR: 12]). The overall 30-day cohort mortality was 65.7% (23 patients).

**Figure 1:**
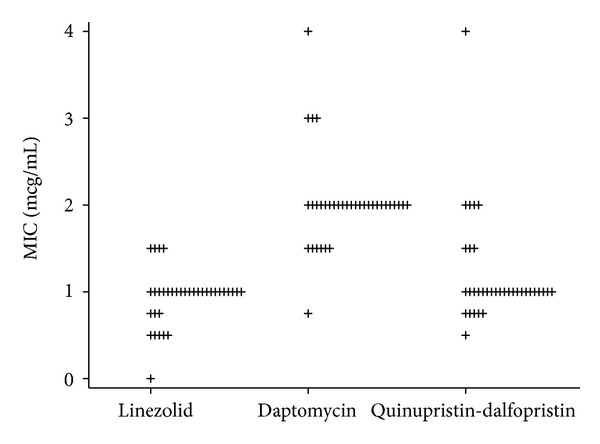
Distribution of specific antibiotic MICs for vancomycinresistant *Enterococcus faecium* isolates. Note: MIC, minimum inhibitory concentration, microgram/mL.

The distribution of specific antibiotic MICs for VREF (Figure 1) shows a favourable *in vitro* susceptibility of all VREF blood isolates to linezolid, daptomycin, and quinupristin-dalfopristin: no case of resistance to these antibiotics was identified.

In the univariate analysis of the factors associated with 30-day mortality (Table 2), treatment with linezolid (*P* < 0.001) was associated with higher survival rates. Presentation with acute kidney injury at the onset of VREF bacteremia was more frequent in nonsurvivors (*P* = 0.002). There was a tendency of association between presence of cirrhosis and the mortality risk (*P* = 0.07). Other variables related to linezolid treatment (e.g., time to antibiotic and duration of treatment) were not associated with the 30-day mortality rate.

After the multivariate Cox proportional hazards model was performed (Table 3, model I), treatment with linezolid was independently associated with a higher survival rate (HR, 0.08; 95% CI, 0.02–0.27), while presence of acute kidney injury at the onset of bacteremia constituted an independent risk factor for 30-day mortality (HR, 4.01; 95% CI, 1.62–9.94). A second multivariate Cox regression model was performed replacing the categorical variable acute kidney injury by the continuous variable initial serum creatinine, while keeping unchanged other variables that reached criteria for entrance in the multivariate analysis (Table 3, model II). This procedure was conducted in order to verify a quantitative relationship between serum creatinine levels and the mortality risk. Each increase of 1.0 mg/dL in the initial serum creatinine level raised the risk of 30-day mortality by 58% (*P* = 0.01).

**Figure 2:**
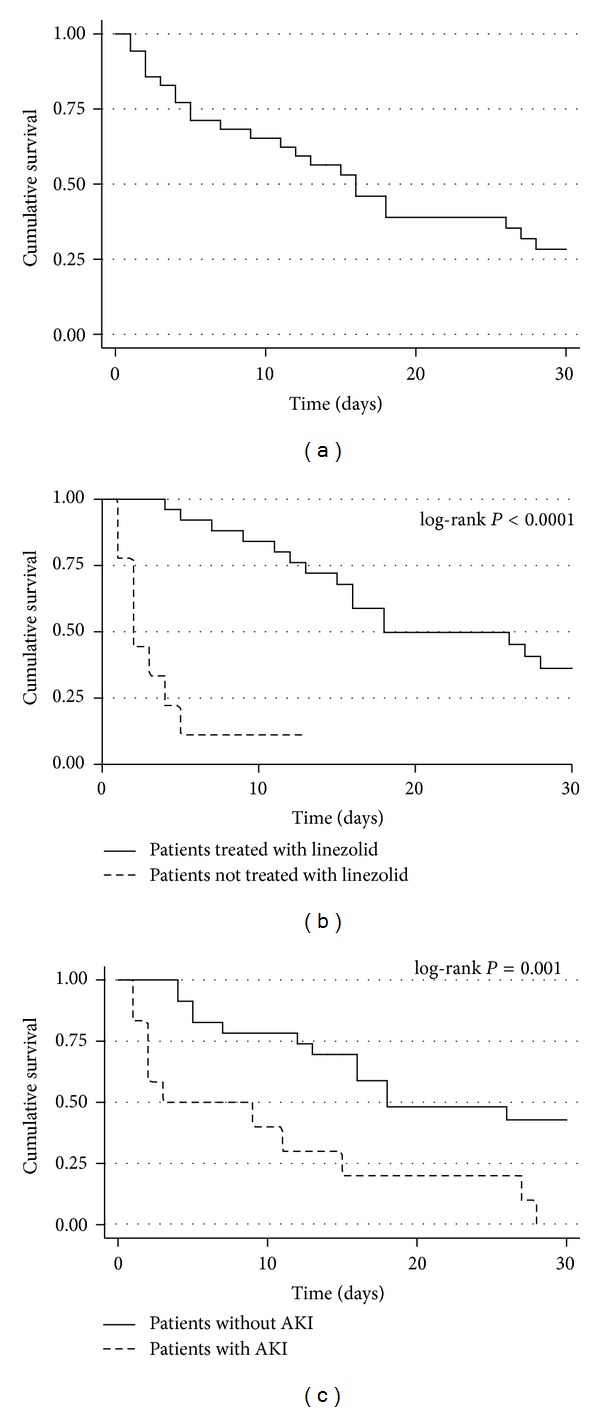
Survival curves of patients with vancomycin-resistant *Enterococcus faecium* (VREF) bacteremia. (a) Survival curve of the entire cohort of patients with VREF bacteremia. (b) Comparison of survival curves of patients treated with linezolid and those treated with other antibiotics without *in vitro* activity against VREF. (c) Comparison of survival curves of patients who presented with acute kidney injury (AKI) at the onset of VREF bacteremia with those who did not present with AKI.

The survival curves of the entire cohort according to linezolid treatment and the presence of acute kidney injury at the onset of bacteremia are shown in Figure 2.

## 4. Discussion

*Enterococcus *is the third most common cause of nosocomial bloodstream infection. VRE is an important problem in Europe, USA, and Latin America and has been isolated in many other countries. Infections due to VRE have been shown to be associated with significant in-hospital mortality and morbidity. Although VRE was first isolated in 1986, the percentage of nosocomial enterococci with vancomycin resistance increased 20-fold in the last 20 years especially among patients in intensive care units, with reported rates of vancomycin resistance varying internationally from 0% to 35% [16, 17]. Despite the fact that 85–90% of clinical isolates of enterococci are *E*. *faecalis*, most VRE are *E*. *faecium* [2]. Similarly, in our institution, the vast majority of VRE bacteremia cases are caused by *E. faecium*. The present study showed a significant incidence of VREF bacteremia among patients with solid and hematologic malignancies as previously described in other studies [9, 10, 18, 19]. Moreover, VREF bacteremia compromised mostly ICU patients with high APACHE II scores, a fact that underscores the relevance of VREF infections in critically ill patients. Although resistance to linezolid, daptomycin, and quinupristin-dalfopristin has been reported in VREF isolates [20, 21], our VREF isolates remained highly susceptible to these antibiotics.

Presentation with acute kidney injury at the onset of VREF bacteremia was more frequent in nonsurvivors. This association has been previously suggested only in studies that have been limited by small numbers of patients and a failure to perform multivariate analysis [19, 22]. Additionally, previous reports estimated renal function solely from blood urea nitrogen and creatinine levels, whereas we used the creatinine clearance, a more physiological estimate of renal function.

Our overall 30-day cohort mortality of 66% was comparable with published data, which range from 17% to 100% [23]. The attributable mortality could not be assessed considering that our study did not perform a case control matched analysis with patients without VREF bacteremia. The survival rate was mainly a result of specific therapy against *E. faecium*. Even with the previous data showing a low bactericidal activity of the oxazolidinone antimicrobial agent against VRE [24, 25], in the present study, linezolid was proved to be an effective therapy against VREF bacteremia in a setting of high prevalence of immunocompromised hosts. Interestingly, time to antibiotic use and duration of antibiotic therapy did not play an important role in the main outcome of our patients.

The retrospective analysis of a relative small cohort of patients is the major limitation of our study considering that we cannot be certain that we have identified all potential confounding factors.

## 5. Conclusions

In summary, our data provide further evidence that VREF is an important cause of mortality in critically ill patients especially with solid and hematological malignancies and renal failure in the ICU setting of a tertiary care institution in Latin America. Despite broad susceptibility to the alternative antimicrobial agents including linezolid and daptomycin against VREF, therapy with ineffective agents for VREF blood stream infections contributed to the poor outcome of the patients.

## Figures and Tables

**Table 1:** Clinical characteristics of 35 patients with bloodstream infection by vancomycin-resistant* Enterococcus faecium*.

Age, years, median (IQR)	46.0 (32.0)
Female sex	14 (40.0)
Type of underlying disease	
Hematologic malignancy	9 (25.7)
Solid malignancy	7 (20.0)
Cirrhosis	4 (11.4)
Diabetes mellitus	3 (8.5)
Connective tissue disease	2 (5.7)
Chronic obstructive pulmonary disease	1 (2.8)
Others*	9 (25.7)
APACHE II score, median (IQR)	26 (10)
Initial plasma CRP, mg/L, median (IQR)	128.5 (177.0)
Initial serum albumin, g/L, median (IQR)	2.4 (1.0)
Acute kidney injury at the onset of bacteremia	12 (34.2)
ICU-acquired bloodstream infection	22 (62.8)

Data presented as *n* (%) unless otherwise indicated. IQR: interquartile range (P75–P25); CRP: C-reactive protein; ICU: intensive care unit. *Others include isolated cases of heart failure, abdominal aortic aneurysm, acute mesenteric ischemia, ischemic stroke, spinal cord injury, vesicorectal fistula, necrotizing fasciitis, cytomegalovirus colitis, and spontaneous pneumothorax.

**Table 2:** Univariate Cox regression analysis of risk factors for 30-day mortality in patients with vancomycin-resistant *Enterococcus faecium* bacteremia.

Variable	Mortality group (*n* = 23)	Survival group (*n* = 12)	HR (95% CI)	*P* value
Age, years, median (IQR)	49 (35.0)	44 (31.5)	1.01 (0.99–1.03)	0.23
Hematologic malignancy	7 (30.4)	2 (16.6)	1.33 (0.54–3.29)	0.52
Solid malignancy	4 (17.4)	3 (25.0)	0.64 (0.21–1.92)	0.43
Cirrhosis	4 (17.4)	0 (0)	2.72 (0.91–8.15)	0.07
Diabetes mellitus	2 (8.7)	1 (8.3)	1.10 (0.25–4.72)	0.89
Chronic obstructive pulmonar disease	1 (4.3)	0 (0)	3.28 (0.41–25.9)	0.25
APACHE II score, median (IQR)	26 (10)	28 (4)	0.97 (0.91–1.04)	0.52
Initial plasma CRP, mg/L, median (IQR)	121.3 (92.2)	222.2 (321.9)	0.99 (0.99-1.00)	0.21
Initial serum albumin, g/L, median (IQR)	2.3 (1.1)	2.8 (0.8)	0.74 (0.29–1.91)	0.54
Acute kidney injury	11 (47.8)	1 (8.3)	3.65 (1.58–8.41)	0.002
ICU-acquired bacteremia	17 (73.9)	5 (41.6)	1.84 (0.72–4.69)	0.19
Linezolid treatment	15 (65.2)	11 (91.6)	0.09 (0.33–0.29)	<0.001
Linezolid MIC, microgram/mL, mean ± SD	0.95 ± 0.28	0.97 ± 0.22	0.78 (0.20–2.96)	0.72
Time to linezolid treatment, days, median (IQR)	2.5 (1.0)	4.0 (5.0)	0.77 (0.57–1.05)	0.11
Duration of linezolid treatment, days, median (IQR)	9.5 (6)	10.5 (10)	0.90 (0.80–1.02)	0.11

Data presented as *n* (%) unless otherwise indicated. HR: hazard ratio; 95% CI: 95% confidence interval; IQR: interquartile range (P75–P25); CPR: C-reactive protein; ICU: intensive care unit; MIC: minimum inhibitory concentration; SD: standard deviation.

**Table 3:** Multivariate Cox regression analysis of factors associated with 30-day mortality in patients with vancomycin-resistant *Enterococcus faecium* bacteremia.

Variable	Adjusted HR	95% CI	*P* value
Model I			
Linezolid treatment	0.08	0.02–0.27	<0.001
Acute kidney injury	4.01	1.62–9.94	0.003

Model II			
Linezolid treatment	0.13	0.03–0.47	0.002
Initial serum creatinine, g/dL	1.58	1.09–2.29	0.01

HR: hazard ratio; 95% CI: 95% confidence interval.

## References

[1] Uttley AHC, Collins CH, Naidoo J, George RC (1988). Vancomycin-resistant enterococci. *The Lancet*.

[2] Murray BE (2000). Vancomycin-resistant enterococcal infections. *The New England Journal of Medicine*.

[3] Edmond MB, Ober JF, Weinbaum DL (1995). Vancomycin-resistant *Enterococcus faecium* bacteremia: risk factors for infection. *Clinical Infectious Diseases*.

[4] Peel T, Cheng AC, Spelman T, Huysmans M, Spelman D (2012). Differing risk factors for vancomycin-resistant and vancomycin-sensitive enterococcal bacteraemia. *Clinical Microbiology and Infection*.

[5] Iosifidis E, Evdoridou I, Agakidou E (2013). Vancomycin-resistant *Enterococcus* outbreak in a neonatal intensive care unit: epidemiology, molecular analysis and risk factors. *American Journal of Infection Control*.

[6] Kang Y, Vicente M, Parsad S (2013). Evaluation of risk factors for vancomycin-resistant *Enterococcus* bacteremia among previously colonized hematopoietic stem cell transplant patients. *Transplant Infectious Disease*.

[7] Hayakawa K, Marchaim D, Martin ET (2012). Comparison of the clinical characteristics and outcomes associated with vancomycin-resistant *Enterococcus faecalis* and vancomycin-resistant E. *faecium* bacteremia. *Antimicrobial Agents and Chemotherapy*.

[8] McKinnell JA, Patel M, Shirley RM, Kunz DF, Moser SA, Baddley JW (2011). Observational study of the epidemiology and outcomes of vancomycin-resistant Enterococcus bacteraemia treated with newer antimicrobial agents. *Epidemiology and Infection*.

[9] Kraft S, Mackler E, Schlickman P, Welch K, DePestel DD (2012). Outcomes of therapy vancomycin-resistant enterococcal bacteremia in hematology and bone marrow transplant patients. *Supportive Care in Cancer*.

[10] Vydra J, Shanley RM, George I (2012). Enterococcal bacteremia is associated with increased risk of mortality in recipients of allogeneic hematopoietic stem cell transplantation. *Clinical Infectious Diseases*.

[11] Kraft S, Mackler E, Schlickman P, Welch K, DePestel DD (2012). Outcomes of therapy vancomycin-resistant enterococcal bacteremia in hematology and bone marrow transplant patients. *Supportive Care in Cancer*.

[12] Twilla JD, Finch CK, Usery JB, Gelfand MS, Hudson JQ, Broyles JE (2012). Vancomycin-resistant Enterococcus bacteremia: an evaluation of treatment with linezolid or daptomycin. *Journal of Hospital Medicine*.

[13] Facklam RR, Collins MD (1989). Identification of *Enterococcus* species isolated from human infections by a conventional test scheme. *Journal of Clinical Microbiology*.

[14] Willey BM, Kreiswirth BN, Simor AE (1992). Detection of vancomycin resistance in Enterococcus species. *Journal of Clinical Microbiology*.

[15] Endtz HP, Van Den Braak N, Van Belkum A (1998). Comparison of eight methods to detect vancomycin resistance in enterococci. *Journal of Clinical Microbiology*.

[16] Leclercq R, Derlot E, Duval J, Courvalin P (1988). Plasmid-mediated resistance to vancomycin and teicoplanin in *Enterococcus faecium*. *The New England Journal of Medicine*.

[17] Iwen PC, Kelly DM, Linder J, Hinrichs SH, Dominguez EA, Patil KD (1997). Change in prevalence and antibiotic resistance of *Enterococcus* species isolated from blood cultures over an 8-year period. *Antimicrobial Agents and Chemotherapy*.

[18] Meyers BR (1996). Nosocomial infections with vancomycin-resistant Enterococcus faecium in liver transplant recipients: risk factors for acquisition and mortality. *Clinical Infectious Diseases*.

[19] Zaas AK, Song X, Tucker P, Perl TM (2002). Risk factors for development of vancomycin-resistant enterococcal bloodstream infection in patients with cancer who are colonized with vancomycin-resistant enterococci. *Clinical Infectious Diseases*.

[20] Bonora MG, Solbiati M, Stepan E (2006). Emergence of linezolid resistance in the vancomycin-resistant *Enterococcus faecium* multilocus sequence typing C1 epidemic lineage. *Journal of Clinical Microbiology*.

[21] Sabol K, Patterson JE, Lewis JS, Owens A, Cadena J, Jorgensen JH (2005). Emergence of daptomycin resistance in *Enterococcus faecium* during daptomycin therapy. *Antimicrobial Agents and Chemotherapy*.

[22] Chou C-H, Lee N-Y, Lee H-C, Chang C-M, Lee C-C, Ko W-C (2012). Emergence of vancomycin-resistant Enterococcus bloodstream infections in southern Taiwan. *Journal of Microbiology, Immunology and Infection*.

[23] Edmond MB, Ober JF, Dawson JD, Weinbaum DL, Wenzel RP (1996). Vancomycin-resistant enterococcal bacteremia: natural history and attributable mortality. *Clinical Infectious Diseases*.

[24] Huang Y-T, Liao C-H, Teng L-J, Hsueh P-R (2008). Comparative bactericidal activities of daptomycin, glycopeptides, linezolid and tigecycline against blood isolates of Gram-positive bacteria in Taiwan. *Clinical Microbiology and Infection*.

[25] Smith PF, Booker BM, Ogundele AB, Kelchin P (2005). Comparative in vitro activities of daptomycin, linezolid, and quinupristin/dalfopristin against Gram-positive bacterial isolates from a large cancer center. *Diagnostic Microbiology and Infectious Disease*.

